# Cerebrovascular pathology in Down syndrome and Alzheimer disease

**DOI:** 10.1186/s40478-017-0499-4

**Published:** 2017-12-01

**Authors:** Elizabeth Head, Michael J. Phelan, Eric Doran, Ronald C. Kim, Wayne W. Poon, Frederick A. Schmitt, Ira T. Lott

**Affiliations:** 1 University of Kentucky, Sanders-Brown Center on Aging, 800 South Limestone Street, Lexington, KY 40536 USA; 2 Department of Statistics, University of California, Irvine, Irvine, CA USA; 3 Department of Pediatrics, University of California, Irvine, Irvine, CA USA; 4 Department of Pathology, University of California, Irvine, Irvine, CA USA; 5 Institute for Memory Impairments and Neurological Disorders, University of California, Irvine, Irvine, CA USA; 6 Department of Neurology, University of California, Irvine, Irvine, CA USA; 7 Department of Neurology, University of Kentucky, Lexington, KY USA

**Keywords:** Arteriolosclerosis, Atherosclerosis, Cerebral amyloid angiopathy, Trisomy 21, Vascular risk factors

## Abstract

People with Down syndrome (DS) are at high risk for developing Alzheimer disease (AD) with age. Typically, by age 40 years, most people with DS have sufficient neuropathology for an AD diagnosis. Interestingly, atherosclerosis and hypertension are atypical in DS with age, suggesting the lack of these vascular risk factors may be associated with reduced cerebrovascular pathology. However, because the extra copy of APP leads to increased beta-amyloid peptide (Aβ) accumulation in DS, we hypothesized that there would be more extensive and widespread cerebral amyloid angiopathy (CAA) with age in DS relative to sporadic AD. To test this hypothesis CAA, atherosclerosis and arteriolosclerosis were used as measures of cerebrovascular pathology and compared in post mortem tissue from individuals with DS (*n* = 32), sporadic AD (*n* = 80) and controls (*n* = 37). CAA was observed with significantly higher frequencies in brains of individuals with DS compared to sporadic AD and controls. Atherosclerosis and arteriolosclerosis were rare in the cases with DS. CAA in DS may be a target for future interventional clinical trials.

## Introduction

People with Down syndrome (DS) are at higher risk for developing Alzheimer disease (AD), which is thought to be primarily due to the overexpression of amyloid precursor protein [19, 46]. Beta-amyloid (Aβ) plaques and neurofibrillary tangles are typically observed by 40 years of age (reviewed in [27], with dementia onset most typically occurring almost a decade later [26, 35, 36, 52]. Up to 55% of people with DS between 40 and 49 years of age develop dementia and the numbers rise to 77% in people 60–69 years of age (reviewed in [6]).

There is increasing recognition of the vascular contribution to cognitive impairment and dementia (VCID; [33, 37]). The presence of cerebrovascular pathology may be a critical comorbidity that accelerates the age of onset of dementia and also leads to faster disease progression. In the general population, ~6%–45% of autopsy cases show mixed AD neuropathology with cerebrovascular pathology [32]. Cerebrovascular pathology resulting from atherosclerosis and arteriolosclerosis may serve as a “second hit” necessary for conversion to dementia particularly when significant Aβ is present in the brain [33, 47]. Atherosclerosis is thought to be a major contributor to VCID as is arteriolosclerosis [1, 29, 33]. Cerebral amyloid angiopathy (CAA) is also observed frequently in AD [3, 15, 25, 38] and may lead to microhemorrhages and infarcts [57].

VCID in DS has been less well studied [60] but is thought to be rare based upon fewer vascular risk factors being present in DS (e.g. hypertension, atherosclerosis, smoking) [45]. In a recent neuroimaging study using T2*and susceptibility weighted imaging, the location and number of microbleeds were evaluated in 91 nondemented and 26 individuals with DS [14]. CAA in people with DS was present in 31% of symptomatic DS participants, which was similar to that observed in sporadic AD (38%). Microbleeds observed by neuroimaging may be larger than the smaller bleeds that can be observed at autopsy and may underrepresent the extent of this vascular pathology in DS. At autopsy, CAA has been reported in small autopsy studies of people with DS over the age of 55 years [30, 35] or in case reports [8, 18, 40] with CAA also containing post-translationally modified Aβ [23]. However, the accumulation of CAA as a function of age in DS has yet to be explored. Extensive cerebrovascular hemorrhages and stroke are associated with CAA in DS [8, 18, 31, 39, 40, 43] in most studies but not in all [30, 35]; the majority of these studies are based on small autopsy series or case reports. Despite increased CAA in DS with age, VCID (or multi-infarct dementia as it was termed then [16]) is rare with only one case report in the literature of a 55-year old woman with DS.

It is important to note that people with DS show virtually no evidence for several of the risk factors for cerebrovascular pathology particularly atherosclerosis and hypertension [12, 20, 21, 41, 42, 61]. Thus, the purpose of our study was two-fold. First, we sought to characterize the frequency of cerebrovascular pathology (defined as atherosclerosis, arteriolosclerosis and CAA) in a series of autopsy cases with DS and AD with direct comparison to sporadic AD and nondemented controls. Second, we hypothesized that cerebrovascular pathology associated with atherosclerosis would be less frequent in DS relative to non-DS AD cases. It is interesting to note that these vascular pathologies have not been directly compared in DS and AD cases.

## Methods and methods

This study examined prevalence of cerebrovascular pathology in three University of California at Irvine Alzheimer Disease Research Center (UCI ADRC) cohorts including DS cases, sporadic AD cases and nondemented control cases. All AD cases, sporadic AD and DS with AD (Braak neurofibrillary tangle (NFT) stage VI and amyloid plaque stage C), were selected based upon neuropathological criteria collected using the recently revised National Alzheimer Disease Coordinating Center (NACC) guidelines [28]. DS cases were all demented at the time of death based upon DSM-IV criteria [56] Atherosclerosis was evaluated using procedures described by Beach and colleagues (2007) [7] with the Circle of Willis being graded by gross visual inspection and rated as none, mild (0–25% obstruction), moderate (26–60% obstruction) or severe (> 60% obstruction). Arteriolosclerosis was determined by H&E staining of the thalamus and subthalamic nuclei, basal ganglia, middle frontal gyrus, superior & middle temporal gyrus, inferior parietal lobule, and occipital cortex [28]. Arteriolosclerosis, as described by Grinberg and colleagues (2010) included concentric hyaline thickening of small arteries (40–150 μm in diameter) associated with a concentric stenosis of the vessel lumen [25]. Arteriolosclerosis was noted as being mild, moderate or severe based upon the severity of hyalinosis of the media and adventitia of small parenchymal and/or leptomeningeal vessels. CAA was scored based upon Aβ immunostaining as none, mild, moderate or severe. Nondemented control cases were selected to be Braak NFT stage 0, I or II and amyloid plaque stage 0 or A [11]. Data collected using the NACC vascular pathology forms at the UCI ADRC describing atherosclerosis, arteriolosclerosis and amyloid angiopathy (or CAA) were used [28]. A total of 149 cases were evaluated, including 32 from the DS cohort, 80 from the AD cohort, and 37 control cases. Demographic characteristics are shown by cohort in Table 1. Similar numbers of males and females were observed in the DS and AD cohorts, but more males were represented in the control cohort. The average age at death was lowest in the DS cohort, and highest in the control cohort (Table 1).

**Table 1:** Characteristics of participants by cohort

	Cohort		
Characteristic^a^	AD (*n* = 80)	DS (*n* = 32)	Ctrl (*n* = 37)
Age-at-autopsy (yrs)	76.08 (12.21)	55.23 (6.64)	80.27 (10.04)
Sex			
Male	39 (49%)	16 (50%)	22 (59%)
Female	40 (50%)	15 (47%)	15 (41%)
Missing	1 (1.2%)	1 (3.1%)	0 (0%)

^a^Means (standard deviation) calculated on a complete-case basis. Female shows the number (percent) of females in the sample. There were two cases with missing entries for their sex

### Relative risk analysis

To determine the relative risk of each of the cerebrovascular outcome measures for the three cohorts, pathological outcomes were dichotomized to indicate the presence/absence of each cerebrovascular event. The risk of a positive finding was then calculated for each cohort, separately. Ninety-five percent confidence intervals were based on the score test for binomial outcomes [2]. Two AD cases were missing measures of atherosclerosis but other measures were available, thus they were excluded from the analysis when comparing atherosclerosis across groups.

### Regression analysis

To test the hypothesis that the DS cohort was less likely to have atherosclerosis or arteriolosclerosis but more likely to have CAA than sporadic AD and control cases, we used multinomial logistic regression. Atherosclerosis, arteriolosclerosis and CAA were graded on an ordinal scale: none, mild, moderate to severe. Cumulative logistic regression was used to model the probability of the severity of CAA findings based on the autopsy case cohort [2]. The cumulative-logit models thus represent the full range of severity and allow for tests of whether each of the cohorts was associated with progressive degrees of severity for each of the cerebrovascular outcomes.

All computations were executed in the graphical and programming environment R [48].

## Results

**Fig. 1:**
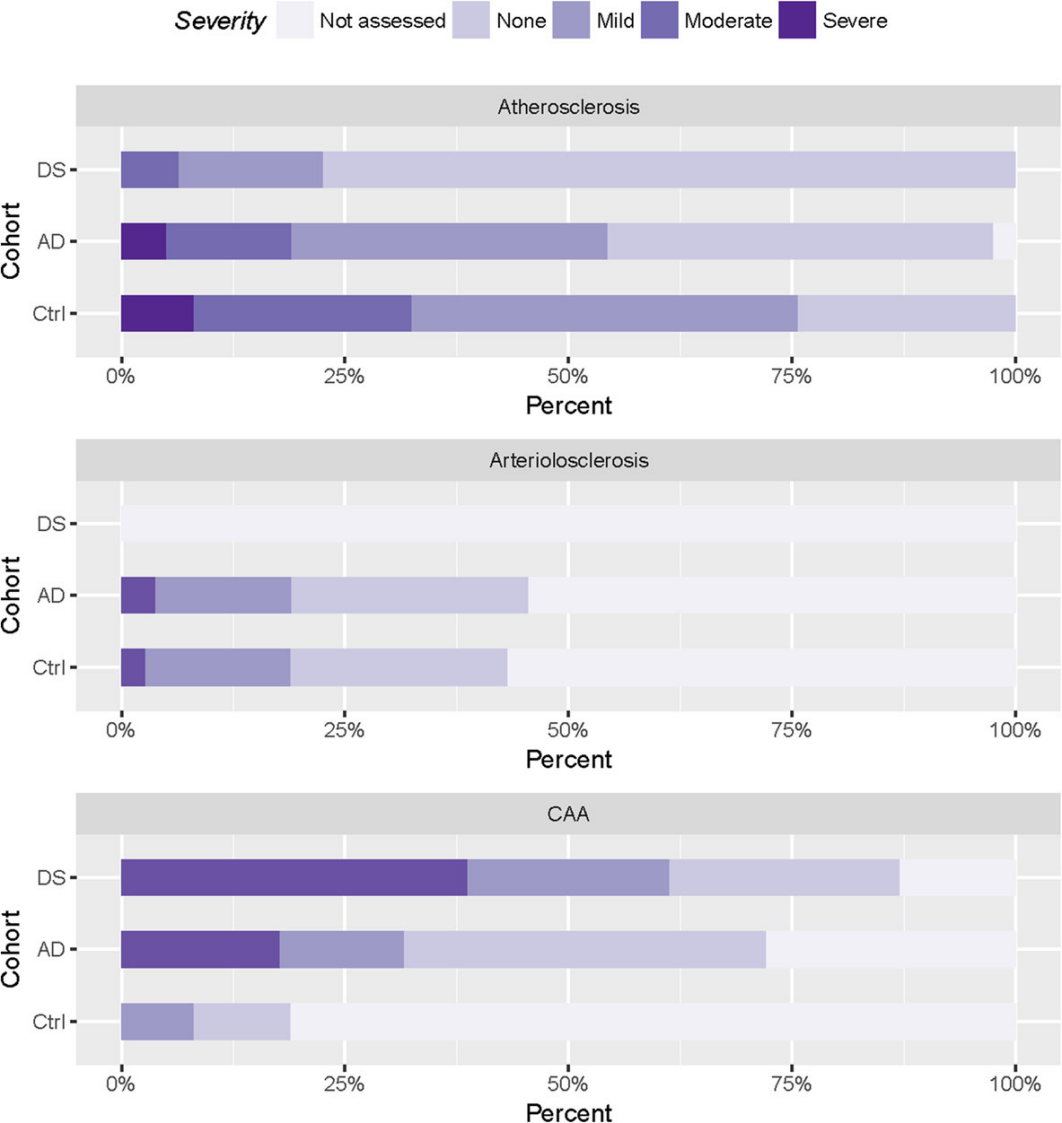
Percentage of autopsy cases by severity of cerebrovascular outcome and cohort. Percentages for Atherosclerosis, Arteriolosclerosis, and Cerebral Amyloid Angiopathy (CAA) add to 100% within each cohort, separately. More moderate and severe CAA findings were associated particularly with the DS cohort

Observations had been recorded using the NACC data forms [28] by a neuropathologist (RK). Atherosclerosis and arteriolosclerosis were frequently observed in the AD cohort and less so in the DS and control cohorts (Table 2, Fig 1). In contrast, CAA was more common in the DS cohort (Fig. 1). The relative risks of each of these vascular outcomes are provided in Table 3 and suggest that the DS cohort was 1.21 times more likely to have CAA relative to AD cases, and 4.6 times more likely to have CAA compared to control cases.

**Table 2:** NACC cerebrovascular outcomes by cohort showing the frequency and percentages for each level of severity

	Cohort		
Characteristic	AD (*n* = 80)	DS (*n* = 32)	Ctrl (*n* = 37)
Atherosclerosis			
Not assessed	2 (2.5%)	0 (0%)	0 (0%)
None	34 (42%)	24 (75%)	9 (24%)
Mild	28 (35%)	5 (16%)	16 (43%)
Moderate	11 (14%)	2 (6.2%)	9 (24%)
Severe	4 (5%)	0 (0%)	3 (8.1%)
Missing	1 (1.2%)	1 (3.1%)	0 (0%)
Arteriolosclerosis			
Not assessed	0 (0%)	0 (0%)	0 (0%)
None	43 (54%)	31 (97%)	21 (57%)
Mild	21 (26%)	0 (0%)	9 (24%)
Moderate	12 (15%)	0 (0%)	6 (6%)
Severe	3 (3.8%)	0 (0%)	1 (2.7%)
Missing	1 (1.2%)	1 (3.1%)	0 (0%)
CAA			
Not assessed	0 (0%)	0 (0%)	0 (0%)
None	22 (28%)	4 (12%)	30 (81%)
Mild	32 (40%)	8 (25%)	4 (11%)
Moderate	11 (14%)	7 (22%)	3 (8.1%)
Severe	14 (18%)	12 (38%)	0 (0%)
Missing	1 (1.2%)	1 (3.1%)	0 (0%)

**Table 3:** Relative risk of cerebrovascular outcome for participants with pairwise comparisons among the cohorts. Each outcome was dichotomized to indicate the presence versus absence of the finding. Relative risk was calculated and reported with the 95% confidence based on the score test for binomial outcomes

DS vs AD			Relative Risk^a^
Cerebrovascular outcome	AD (*n* = 80)	DS (*n* = 32)	Estimate (95% CI)
Atherosclerosis	43 (55.84%)	7 (22.58%)	0.40 (0.20, 0.74)
Arteriolosclerosis	36 (45.57%)	0 (0.00%)	0.00 (0.00, 0.24)
CAA	57 (72.15%)	27 (87.10%)	1.21 (0.96, 1.46)
DS vs Ctrl			Relative Risk
	Ctrl (*n* = 37)	DS (*n* = 32)	Estimate (95% CI)
Atherosclerosis	28 (75.68%)	7 (22.58%)	0.30 (0.15, 0.55)
Arteriolosclerosis	16 (43.24%)	0 (0.00%)	0.00 (0.00, 0.26)
CAA	7 (18.92%)	27 (87.10%)	4.60 (2.49, 9.29)
AD vs Ctrl			Relative Risk
	Ctrl (*n* = 37)	AD (*n* = 80)	Estimate (95% CI)
Atherosclerosis	28 (75.68%)	43 (55.84%)	0.74 (0.56, 0.99)
Arteriolosclerosis	16 (43.24%)	36 (45.57%)	1.05 (0.70, 1.68)
CAA	7 (18.92%)	57 (72.15%)	3.81 (2.07, 7.70)

^a^Cerebrovascular outcomes were dichotomized for the presence vs the absence of positive finding. Relative risk compares DS to AD, DS to Control and AD to Control. The confidence intervals were calculated using the score test for the binomial distribution

To test the hypothesis that the DS cohort had a higher frequency of more severe CAA and a lower frequency of severe atherosclerosis or arteriolosclerosis, we used a cumulative logistic regression analysis. Based on a χ^2^ test with 2 degrees of freedom, there was a significant association of CAA with the type of autopsy cohort (Likelihood ratio statistic 47.96, *p* < .0001). Cumulative logistic regression may be used to compare the odds of a higher-versus-lower severity finding between cohorts based on the odds ratio. According to Table 4, the odds of severe CAA in the DS cohort was approximately three times the odds of a higher severity finding in the AD cohort (odds ratio = 3.05 (95% CI 1.4, 6.67), *p* = 0.005). Arteriolosclerosis, in contrast, was absent in the DS cohort, and there were no significant differences between the AD and control cohorts. Atherosclerosis was significantly less severe in the DS cases relative to the AD (odds ratio = 0.23, (95% CI 0.09, 0.59), *p* = 0.0026) and control cases (odds ratio = 0.11, (95% CI 0.04, 0.30), *p* < .0001).

**Table 4:** Pairwise comparisons among cohorts for each of the vascular outcome measures. The odds ratio (OR) of a higher-level finding was compared among cohorts. Estimated odds used cumulative logistic regression to model the chance of higher-versus-lower severity outcome based on an indicator of the cohort. For arteriolosclerosis, only the AD versus Ctrl comparison could be reliably estimated

Odds Ratios (OR) among Cohorts			
Vascular finding	Est. (95% CI)	*P*-value	df
Atherosclerosis			
AD vs Ctrl	0.46 (0.22, 0.95)	0*.*0353	140
AD vs Ctrl	0.46 (0.22, 0.95)	0.0353	140
DS vs Ctrl	0.11 (0.04, 0.30)	*< *0*.*0001	140
DS vs AD	0.23 (0.09, 0.59)	0*.*0026	140
Arteriolosclerosis			
AD vs Ctrl	1.08 (0.51, 2.32)	0*.*8376	142
CAA			
AD vs Ctrl	10.12 (3.94, 25.96)	*<* 0*.*0001	142
DS vs Ctrl	30.88 (10.15, 93.99)	*< *0*.*0001	142
DS vs AD	3.05 (1.40, 6.67)	0*.*0055	142

### Atherosclerosis, arteriolosclerosis and age in DS, AD and controls

**Fig. 2:**
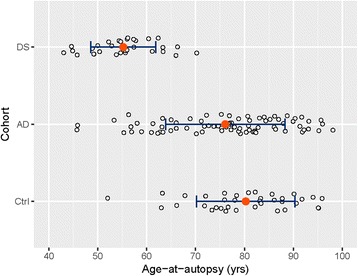
Age at autopsy across the three cohorts (DS, AD, Ctrl) showing that the DS cases were younger than the AD and Control (Ctrl) cases. Individual data points are shown in open circles, group means in solid, red circles. Error bars show plus-and-minus one standard deviation from the mean

**Fig. 3:**
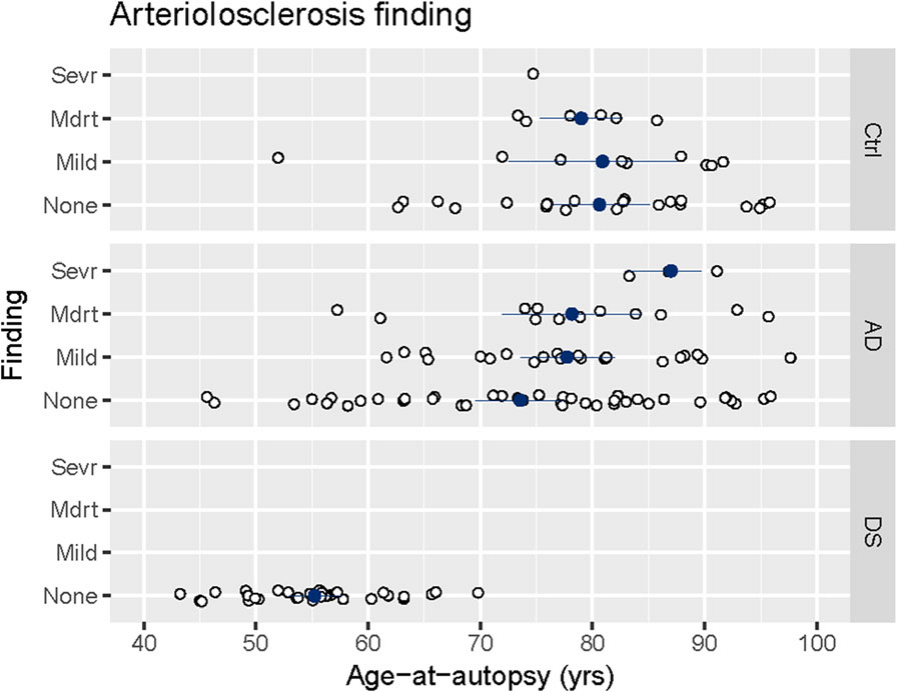
Arteriolosclerosis as a function of age in DS, AD and Controls. Individual data points shown as open circles, group means as filled circles. Error bars show plus-and-minus one standard deviation from the mean

**Fig. 4:**
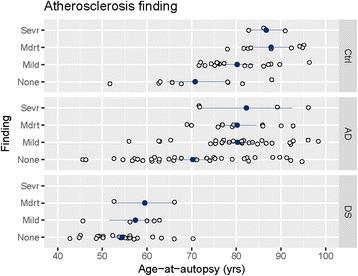
Atherosclerosis as a function of age in DS, AD and Controls. Individual data points shown as open circles, group means as filled circles. Error bars show plus-and-minus one standard deviation from the mean

As shown in Fig. 2, the DS cohort is younger than the AD and control cohorts. The relative youth of the DS cohort may explain the absence of arteriolosclerosis, while control (16/37 = 43%) and AD cases (36/80 = 45%) (Table 2) showed mild to severe arteriolosclerosis (Fig. 3). Similarly the relative youth may explain the low frequency of atherosclerosis in DS cohort (7/31 = 22.6% -Table 2) and these lesions were in the mild to moderate range (Fig. 4), whereas the frequencies in AD and control cohorts were 43/77 (55.8%) and 28/37 (75.7%), respectively (Table 2). However, in cases that were under 70 years of age, only one control case showed mild arteriolosclerosis and 6 sporadic AD cases showed mild to moderate arteriolosclerosis. Atherosclerotic lesions were observed in a subset of DS cases (25.3%) and these lesions were in the mild to moderate range (Fig. 4). To determine whether younger sporadic AD cases and controls show less frequent and severe atherosclerosis comparable to our DS cohort, we separated the bottom 10% of the AD and control cohorts such that they overlapped with the overall age distribution of the DS cohort. This cutoff left 4 control cases and 8 AD cases. The younger controls showed no CAA, no atherosclerosis and only one case had mild arteriolosclerosis. In contrast, one AD case showed mild atherosclerosis, one showed moderate arteriolosclerosis and almost half showed CAA.

### CAA severity increases with age in DS cases

**Fig. 5:**
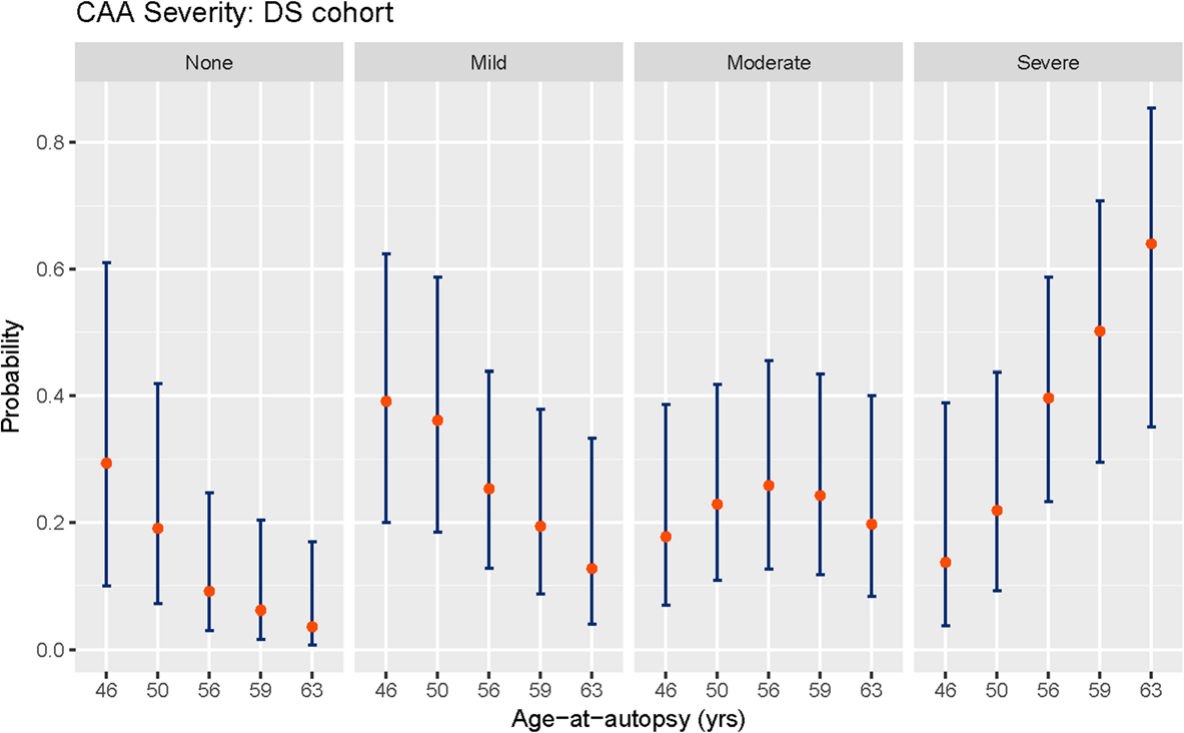
Probability of CAA severity as a function of age-at-autopsy in DS cohort. Error bars show the upper and lower limits of the 95% confidence interval. The number of DS cases with no or mild CAA decreases with age. The number of people with DS with moderate CAA remains relatively stable but the numbers of cases with severe CAA increases with age

Participants in the DS cohort were on average 20 to 25 years younger at autopsy than participants in the AD or Control cohorts (Fig. 2). The strong separation between the groups, that is DS versus AD plus Control, meant that age-at-autopsy and cohort were effectively confounded in any attempt to account for their relative contributions to the observed distribution of CAA. We found it productive, however, to address the question by analysis of the association with age-at-autopsy in each cohort, separately. Our results showed in particular that age-at-autopsy was strongly correlated with CAA severity among DS participants, but not among participants in the AD or the Control cohorts (Fig. 5). Thus, using a cumulative logistic regression model with CAA severity as the dependent variable, we estimated the probability of the severity of CAA based on age at autopsy for each cohort. In each case the null model, excluding age as predictor, was compared to the alternative model, including age as predictor, using a Chi-squared likelihood ratio statistic (LRS) based on 1 degree of freedom (df). The results showed that a trend with age-at-autopsy was strongly associated with CAA finding in the DS cohort at the 5% level of significance (LRS = 6, df = 1, *P*-value = 0.014). Again at the 5% level of significance, the results also showed that age-at-autopsy was *not* significantly associated with CAA findings in the AD cohort (LRS = 0.57, df = 1, *P*-value = 0.45) nor in the Control cohort (LRS = 2.43, df = 1, *P*-value = 0.119).

**Fig. 6:**
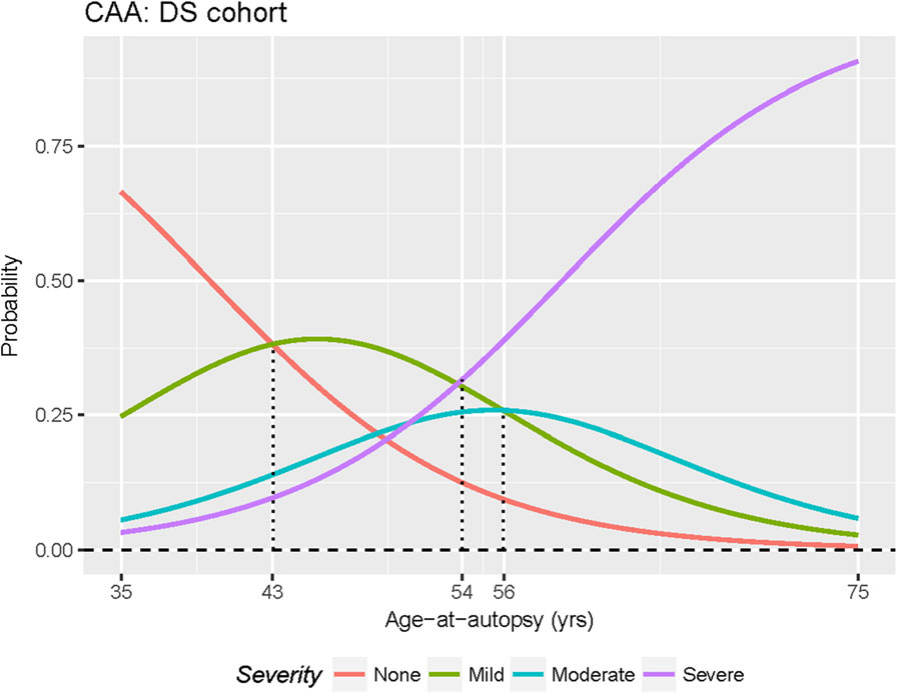
Probability curves for CAA severity based on age-at-autopsy in DS cohort. The age at which CAA shifts from mild, to moderate to severe are estimated based on age. Moderate to severe findings were more likely at upper ages

Figure 6 graphs the estimated probability curves for the DS cohort. The estimated probability of a severe finding is shown as an increasing function of age and becomes the most likely finding at about age 54 years. A finding of ‘none’ is shown as the most likely finding before age 43 years, beyond which the most likely finding was estimated to be ‘mild’, at least until the age of about 54 years. Figure 5 graphs the estimated probabilities of each finding at selected ages, including a 95% confidence interval for the population value. At younger ages, say 46 to 50 years in Fig. 5, the confidence intervals overlap appreciably among the various findings. But at the older ages, say 56 years and above, the intervals separate, and the likelihood of a severe finding tends to dominate. Note that above age 59 years in DS, severe CAA was more common than mild or moderate CAA. In contrast to these results, the estimated probability curves (not shown) were basically flat in the AD and Control cohorts, reflecting the non-significant trends with age-at-autopsy in these groups. Nevertheless, the observed trends in the DS cohort appeared to be of independent interest.

## Discussion and conclusions

We hypothesized that people with DS would have significantly more frequent and more severe CAA associated with overexpression of APP relative to sporadic AD and control cases but less cerebrovascular pathology typically associated with cardiovascular risk factors including atherosclerotic lesions and arteriolosclerosis. In the current study, as expected, we found: (1) the presence of CAA in DS was more frequent than in the AD and control cohorts; (2) that the DS cohort showed an increased probability of more severe CAA with age; and (3) atherosclerosis and arteriolosclerosis vascular pathology was uncommon in DS.

### CAA in controls, AD and DS

The current study observed CAA in 87.1% of DS cases, 18.9% of controls and 72.2% of AD cases. This is significantly higher than a previous report using neuroimaging outcomes to detect microbleeds in a larger cohort [14]. In a separate study, the frequency of CAA in a prospectively followed cohort of nondemented individuals without DS (*n* = 59 cases with an average age of 83.9 years at autopsy) has been reported as high as 62% [17]. In contrast, in a NACC study of 140 nondemented control cases without AD neuropathology with an average age at death of 83.5 years, 7.5% exhibited CAA [9]. The NACC study was consistent with the Medical Research Council of Cognitive Function and Ageing Study in England and Wales where 7% of 109 nondemented control cases (70–103 years) exhibit CAA [44]. In contrast, the Adult Changes in Thought (ACT) study noted that 15.7% of control cases (*n* = 89, 70 years to over 90 years) had CAA [54]. In studies of AD cases, consistently more frequent CAA is observed that range between 37%–83% of autopsy cases [22, 44, 49, 54]. Thus, the range of cases affected by CAA in our AD and control cohorts is not inconsistent with previous reports.

The presence of CAA in 87.1% of DS cases is high both relative to the AD cohort examined here as well as previous reports in nondemented controls and AD cases, suggesting that CAA may be a critical vascular pathology associated with aging in DS. A recent neuroimaging study using T2*-based MR outcome measures observed that CAA was present in 31% of cognitively impaired people with DS [14].

More frequent CAA is observed in autosomal dominant AD subjects, who also exhibit Aβ overproduction and develop early onset AD. In a study by Ringman and colleagues, autosomal dominant AD cases who were of similar age to the current cohort of DS autopsy cases (average age 53.5 years) showed more moderate to severe CAA (63.3%) as compared to older sporadic AD cases (39.2%, mean age 79.3 years) [49]. In contrast, in rare autopsy cases of DS with partial trisomy 21, where APP is not overexpressed, AD neuropathology and CAA was absent even at 72 or 78 years of age at autopsy [19, 46]. Thus, higher levels of CAA in DS are most likely due to APP overexpression and consequent increased Aβ accumulation. CAA in people with DS is more consistent with the CAA prevalence in early onset autosomal dominant AD.

### Arteriolosclerosis and Atherosclerosis in AD, controls and DS

Atherosclerosis and arteriolosclerosis were uncommon in the DS cohort, which may be due to their younger ages (50–59 years). In a NACC study of older nondemented autopsy cases lacking AD pathology, 77.6% of cases showed arteriolosclerosis and 82.9% showed atherosclerosis (*n* = 140, mean age 83.5 years) [9]. In studies of nondemented autopsy cases under 70 years of age (50–70 years), 68.6% (*n* = 1008) were positive for arteriolosclerosis [29]. Further, Ighodaro et al. showed using NACC autopsy data from cases at an average age of 50 years that only 20% exhibited arteriolosclerosis.

### Implications of CAA in DS

The contribution of CAA pathology to the development of AD neuropathology and/or dementia is increasingly recognized as being critical [33, 55]. Further, cerebrovascular lesions may be one of the underlying causes of variable (and modest) clinical trial outcomes as they may be more or less engaged in individual patients and possibly blunting responses to Aβ interventions. In DS, the higher frequency of CAA is likely to lead to several possible outcomes: First, CAA can lead to vascular dysfunction including impaired constriction and dilation, which is consistent with reports of reduced FDG-PET with age in DS and also associated with cognitive decline [51]. Second, CAA can lead to blood brain barrier disruptions and microhemorrhages [50, 58], which also may contribute (in addition to APP overexpression) to the earlier age of onset of dementia in people with DS. Interestingly, a recent review by Buss and colleagues suggests that despite the presence of CAA in DS, there appears to be less intracerebral hemorrhage suggesting possible protective mechanisms; this will be an exciting avenue of research for future studies [13].

It is important to consider some caveats to the current study, which include the use of observational data, the relatively small sample size of our DS cohort and the younger age of this cohort. Autopsy studies of DS are a challenge as the number of tissue donations are relatively small, thus our samples size is smaller relative to sporadic AD cohorts. Further, people with DS who volunteered for this research study and consented to autopsy may also represent a biased sample. Age is virtually impossible to control for in the comparison of these three groups as people with DS have a significantly younger age of onset of AD neuropathology and development of dementia.

The higher frequency of CAA in DS compared to sporadic AD is interesting in that cerebrovascular pathology in DS appears to have a unique signature, i.e. significant CAA and low or absent atherosclerosis and arteriolosclerosis, which could impact cognition and age of onset of dementia in DS. Weller et al. have suggested that CAA may impact therapeutic outcomes and may predispose people to vasogenic edema and hemorrhagic complications due to reduced drainage of Aβ associated with affected vessels [59]. Experimental evidence by the same group shows that periarterial lymphatic drainage is impaired with age and CAA. With an increase in production of Aβ in DS it seems likely that CAA and the presence of Aβ plaques would form at a younger age and with less severity of arteriolosclerosis in DS than in sporadic AD. The present study supports the hypothesis that a life-long increase in production of Aβ in DS predisposes to an earlier onset of age-related impairment of periarterial elimination of Aβ from the brain and thus may accelerate the onset of the pathological features of AD [4] .

While the hypothesis of reduced periarterial elimination of Aβ from the brain is compelling, in DS it does not readily account for findings from other groups of Aβ’s role in cerebrovascular disease and plasma levels of Aβ. For example, Gomis and colleagues show that plasma Aβ1–40 levels are associated with cerebrovascular small vessel disease in acute lacunar stroke and suggest that vascular Aβ is primarily Aβ1–40, which alters endothelial functions [24]. In DS, plasma data show that the risk of dementia in DS is increased as levels of Aβ1–42 decline but Aβ1–40 levels increase [53]. As reviewed by Biffi and Greenberg, a high Aβ 40:42 ratio appears to be an important index of vascular amyloid formation [10]. However, Carmona-Iragui and colleagues did not find differences in CSF Aβ40 that corresponded to microbleeds by neuroimaging in DS [14]. Therefore, investigation of plasma markers and CAA presence in DS as related to clinical dementia may become an important avenue of investigation.

If we extend these findings in the context of considering possible interventions for people with DS to prevent AD, it will be important to target CAA pathology specifically. For example, there is a clinical trial of ponezumab (PF-04360365) that is specifically targeting CAA as a treatment for AD that may be very relevant for the DS population [5, 34]. Thus, CAA pathology in DS may be a significant factor to consider in the design of future clinical trials.

In future, it will be important to link neuropathological vascular findings with clinical outcomes (e.g. severity of dementia in DS) and to further explore the additional downstream pathologies associated with CAA. For example, the role of microhemorrhages in DS and their link to the extent of CAA in DS is as yet unknown. Further it will be interesting in future studies to distinguish CAA that is associated with capillary Aβ and associated CAA findings such as microinfarcts, superficial siderosis, etc. Neuroimaging may also provide novel insights for vascular pathology in DS as has been reported previously [14]. Using T2* magnetic resonance imaging, Carmona-Iragui and colleagues observed CAA in 31% and 38%, respectively in people with DS who were nondemented compared with those with dementia. Linking neuroimaging outcomes to subsequent autopsy studies will be a challenge for studies of DS but should evolve over time.
